# Patient satisfaction after elective implant removal of the lower extremity — a retrospective cohort study

**DOI:** 10.1007/s00068-024-02462-6

**Published:** 2024-02-06

**Authors:** Jan Hambrecht, Claudio Canal, Felix Karl-Ludwig Klingebiel, Paul Köhli, Valentin Neuhaus, Hans-Christoph Pape, Yannik Kalbas, Christian Hierholzer

**Affiliations:** 1 University Hospital Zurich: UniversitatsSpital Zurich, Zurich, Switzerland; 2 Charité - Universitätsmedizin Berlin, Berlin, Germany

**Keywords:** Implant removal, Healed fractures, Lower extremity, PROMs/Patient satisfaction

## Abstract

**Purpose:**

The topic of elective implant removal (IR) in healed fractures of the lower extremity remains controversial, particularly when unspecific symptoms of discomfort, which cannot be quantified, are the primary indication. This study aims to assess indications and outcomes of elective IR of the lower extremity, focusing on unspecific symptoms of discomfort and patient satisfaction postoperatively.

**Materials and methods:**

The retrospective cohort study was conducted at a single level I academic trauma center. We included patients who underwent elective IR for healed fractures of the ankle, foot, patella, and proximal tibia from 2016 to 2021. All patients were followed-up for a minimum of 6 weeks after IR. Our outcomes of interest were patient satisfaction, complications, and alleviation of complaints.

**Results:**

A total of 167 patients were included in the study. Unspecific symptoms of discomfort were the most common reason for IR in all investigated anatomical regions of the lower extremity (47.9%), followed by pain (43.1%) and limited range of motion (4.2%). 4.8% of patients experienced a combination of pain and range of motion limitation. Among all patients, 47.9% reported subjective improvement after IR. IRs based on unspecific symptoms of discomfort were significantly less likely to show alleviation of complaints after IR (27.5%, OR 0.19, *p* ≤ 0.01). Patients who reported limited range of motion (OR 1.7, *p* = 0.41) or pain (OR 6.0, *p* = 0) were significantly more likely to be satisfied after IR. Patients who reported sensitivity to cold weather also showed a decrease of complaints after IR (OR 3.6, *p* = 0.03). Major complications occurred in 2.1% of cases. The minor complication rate was 8.4% (predominantly impaired wound healing). Smoking patients showed a significantly higher risk of complications after IR (OR 5.2, *p* = 0.006). Persistent pain postoperatively was detected in 14.7%.

**Conclusion:**

When elective IR for consolidated fractures of the lower extremity is primarily motivated by patients’ subjective symptoms of discomfort, the risk for postoperative dissatisfaction significantly increases. Objective symptoms on the other hand are associated with higher satisfaction after IR. While the procedure is generally safe, minor complications such as wound healing disorders can occur, especially in smokers. Patient education and well-documented informed consent are critical.

## Purpose

Elective removals of implants following surgical treatment of fractures in the lower extremity are continuously increasing. Roughly, 25% of operatively treated foot and ankle injuries receive IR. Furthermore, up to 30% of all orthopedic surgeries are IRs [1]. Prior studies have demonstrated alleviation of symptoms following IR in patients with symptomatic implants and objective restrictions [2, 3]. However, indications for IR of the lower extremity are diverse. While there are several clear-cut indications such as in revision surgery with re-osteosynthesis for nonunion, peri-implant fracture, implant failure, or infection, other indications include pain, limited range of motion, or unspecific symptoms of discomfort that may vary depending on weather conditions are still controversial [4]. A study from Germany identified pain and limited range of motion as the primary factors leading to IR. In addition, the study outlined common postoperative complications, including wound healing disorders (21%), residual materials (12%), and nerve injuries (14%) [5]. Given the impact of these complications on the daily lives of individuals, particularly in relation to the lower extremity, they can significantly diminish quality of life. However, the scientific literature and guidelines concerning IR are limited. The issue becomes even more controversial when considering unspecific symptoms of discomfort, which are often cited as a reason for IR. Assessing these complaints objectively can be difficult as they primarily rely on the patients’ own descriptions, rendering standardized quantification difficult, while the scientific base for evidence-based decision-making in these cases is scarce [3]. Therefore, we initiated this study to investigate the indications, complications, and patients’ satisfaction following elective IR in fully healed fractures of the lower extremity.

Additionally, this study aims to determine whether there is a variation in outcomes between patients who present with objective complaints and those without after undergoing elective IR. We hypothesized that indications for IR based on unspecific symptoms of discomfort may result in similar patient satisfaction, compared to clinically objective indications, such as pain or limited range of motion.

## Material and methods

### Study design

Retrospective, cross-sectional study design with at least 6 weeks of follow-up. The reporting of this study adheres to the STROBE (Strengthening the Reporting of Observational Studies in Epidemiology) statement [6].

### Ethical considerations

Only patients who consented to give written consent were included. The local ethics committee of the University of Zurich approved this study conducted according to the Declaration of Helsinki (PB_2016-01888, Ethical Committee University Zurich).

### Study populations

Patients who underwent IR of the lower extremity in a period between 2016 and 2021 in an academic level 1 trauma center in Switzerland were included. We specifically screened the electronic clinical information system for patients who visited our outpatient clinic with radiologically healed fractures in the lower extremities following open reduction and internal fixation. We analyzed the four most common anatomical regions for IR of the lower extremity (ankle, foot, proximal tibia, and patella). Patients who had their implants removed in another anatomical region were excluded. All investigated procedures were performed in an ambulatory setting. As IRs of higher-risk patients are typically performed in an inpatient setting at the hospital, those patients were excluded from the study. Furthermore, patients under 18 years of age, patients who did not provide written informed consent, or patients with missing data were also excluded from this study.

### Surgery and follow-up

All patients in this study exhibited radiologically consolidated fractures of the lower extremity at least 1 year after open reduction and internal fixation. The procedures took place in our outpatient clinic in a “same day surgery” setting. General anesthesia was employed for all cases. Subsequently, the patients underwent regular postoperative follow-ups, with a 1-day appointment for wound control and a 6-week appointment for clinical examination and radiological assessment.

Patients underwent evaluation before and after surgery. The assessment of indications for IR involved a clinical examination that considered both subjective and objective criteria. Objective criteria included potential wound healing disturbances and clinical limitations such as triggerable pain or objectively restricted range of motion (ROM), while subjective criteria predominantly encompassed nonspecific symptoms of discomfort. In terms of pain management, patients were queried about the necessity for pain medication. The assessment of patients for IR was determined by the parameters pain, limited range of motion, and unspecific symptoms of discomfort. The primary outcome of interest was overall patient satisfaction, which was assessed during the routine 6-week follow-up after IR. The patient satisfaction was defined as an improved postoperative status with patients experiencing a noticeable relieve of symptoms compared to the preoperative condition. Additional outcome parameters included complications and objective functional improvements. The clinical examination was conducted by a physician of our Trauma Department. We compared the preoperative and postoperative range of motion for the ankle and the foot using the neutral-zero-method. For the ankle joint, ab- and adduction, in- and eversion, dorsal extension, and plantar flexion were measured. For the foot, we measured the movement of the toes (adduction, abduction, flexion, extension). Enhancement in ROM was noted when there was an increase of more than 10° in ROM between the pre- and postoperative measurements.

### Definitions

*Patient satisfaction* was defined as an improved postoperative status with patients experiencing a noticeable or meaningful relieve of symptoms compared to the preoperative condition. *Pain* was described as the need for painkiller and complaints that could be activated. *Limited range of motion* was defined as objective complaints and demonstrated limitations upon clinical examination. *Symptoms of discomfort* were characterized as the discomfort experienced by patients without specific, identifiable complaints or limitations, and in the absence of painkiller usage. *Other* anatomical locations are defined as patella and proximal tibia. Procedures on the *foot* are defined as IRs on the metatarsal, talus, and calcaneus. *Major complications* are defined as injuries of relevant surrounding structures as well as major bleeding or refractures. *Minor complications* are defined as wound healing disorders as well as superficial bleedings.

### Statistical analyses

Continuous data are presented with mean and standard deviation (SD), and categorical variables are presented with numbers and percentages. Statistical analysis was performed in R (R Core Team (2018). R: A language and environment for statistical computing. R Foundation for Statistical Computing, Vienna, Austria. URL: https://www.R-project.org/).

The ggplot2-package was used for data visualization. Data was visually tested for normality using histograms. Unpaired Student *T*-tests were used for parametric data. Non-parametric data was tested using Wilcoxon-Mann–Whitney tests. Binary categorical data was assessed using Fisher’s exact test, and non-binary categorical data using chi-squared test with Yates’ correction for continuity. Odds ratios (OR) were calculated using a logistic regression model. The threshold for statistical significance was determined as a *p*-value of < 0.05.

## Results

### Study population

**Fig. 1:**
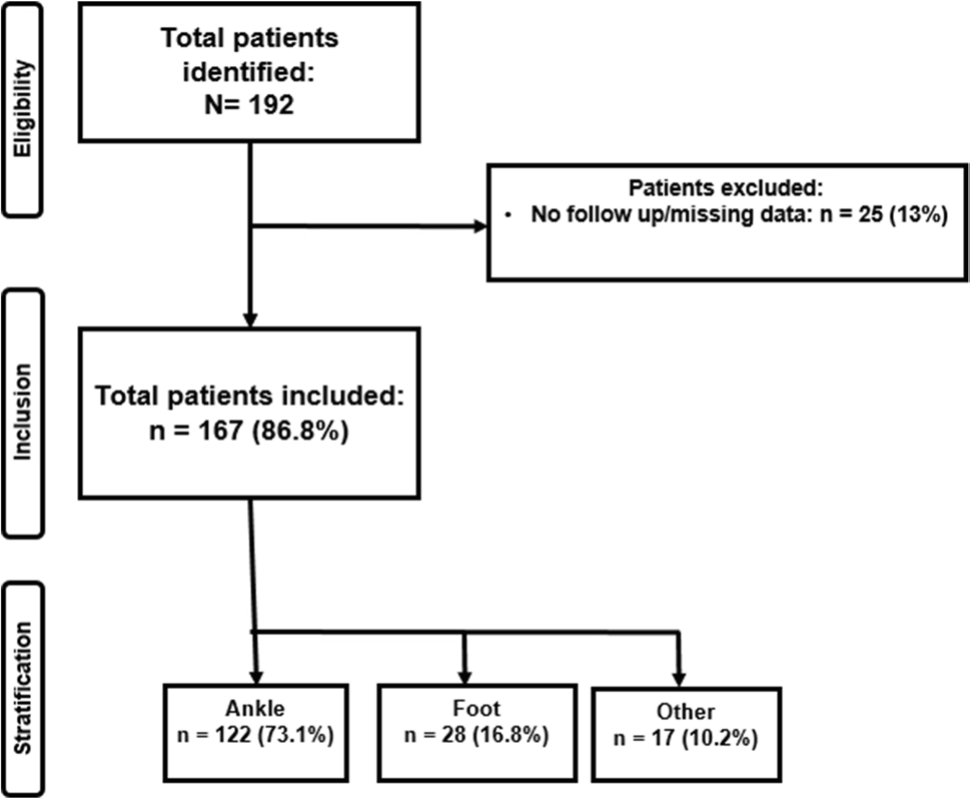
Patient selection flowchart

In total, we encountered 192 patients who underwent elective IR of the lower extremity. However, 25 patients (13%) were excluded because they did not have a complete follow-up. Consequently, 167 patients (86.8%) were included. Out of these, 122 patients (73.1%) underwent IR at the *ankle*, 28 patients (16.8%) had IR of the *foot*, and 17 patients (10.2%) had an IR from *other* regions of the lower extremity (patella and proximal tibia) (Fig. 1).

### Patient demographics and indications for IR

Of the 167 included patients, 71 were female (42.5%) and 96 male (57.5%) with a mean age of 44.7 years (SD = 14.8). Implants were removed on average after 1.2 years. Relevant comorbidities with a risk of wound healing disorders (e.g., diabetes, peripheral artery disease, immunotherapy, kidney failures) were seen in 27% of the patients. Active smoking was reported by 32% of the patients. Patient demographics and indications are presented in Table 1.

**Table 1:** Demographics and indications stratified by body region. Other = patella and proximal tibia

	Ankle	Foot	Other
*n*	122	28	17
Demographics			
Age (mean (SD))	43.75 (± 15.13)	45.29 (± 13.04)	50.24 (± 16.28)
Sex = female (%)	57 (46.7)	10 (35.7)	4 (23.5)
Duration of surgery (mean ± SD)	25.08 (± 16.98)	33.36 (± 21.59)	40.24 (± 13.77)
Smoking	36 (29.5)	12 (42.9)	4 (23.5)
Comorbidities	37 (30.3)	13 (46.4)	3 (17.6)
Indication, *n* (%)			
Pain + limited ROM	4 (3.3)	2 (7.1)	2 (11.8)
Limited ROM	4 (4.3)	2 (7.1)	1 (5.9)
Pain	55 (45.1)	11 (39.3)	6 (35.3)
Subjective	59 (48.4)	13 (46.4)	8 (47.1)

Patients were categorized (ankle, foot, and other) based on their eligibility for IR, determined by factors such as triggerable pain necessitating painkillers, objectively restricted ROM, or nonspecific discomfort symptoms. For IR after osteosynthesis of ankle fractures, we observed unspecific symptoms of discomfort as the predominant indication (48.4%). Pain was reported in 45.1% as the main reason for IR. A limited range of motion without other complaints was found in 3.3%. A combination of pain and limited range of motion was detected in 3.3%.

**Fig. 2:**
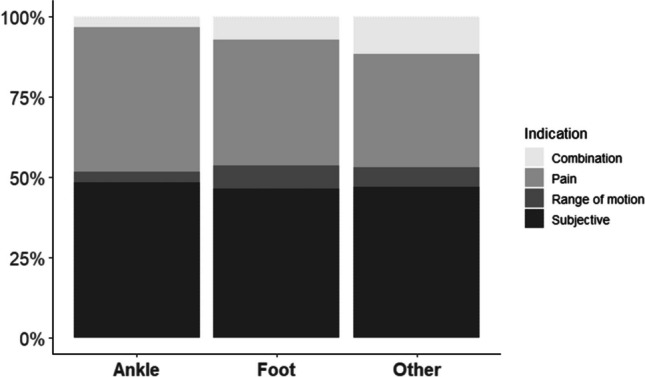
Indications for implant removal stratified by anatomical region

Regarding IR on the foot, we identified symptoms of discomfort as the most common reason for IR (46.4%). Pain was reported in 39.3% and limitation in range of motion in 7.1% of the patients. The combination of pain and limited range of motion was found in 7.1% as well. For other fractures of the lower extremity, including IR of the patella or proximal tibia, the leading indication for IR was also unspecific symptoms of discomfort (47.1%). The secondary reason for IR was pain (35.3%). Pain and limited range of motion were observed in 11.8% of the patients. Limited range of motion was observed as the main indication in 5.9% as presented in Table 1 and Fig. 2***.*** A sensitivity to cold weather was only observed in ankle fractures (9.8%) and fractures of the foot (14.3%).

### Patient satisfaction

**Fig. 3:**
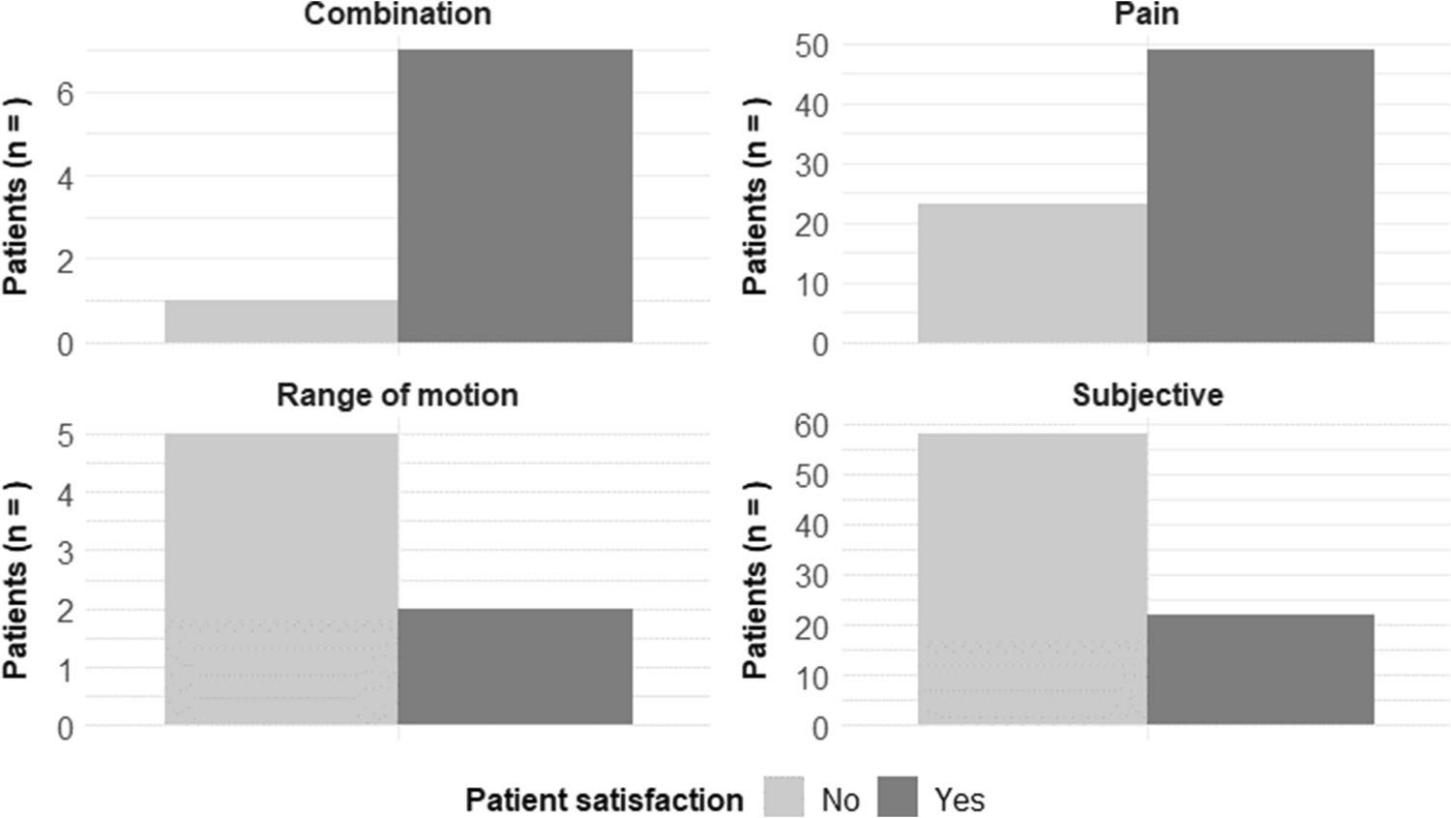
Patient satisfaction after implant removal in view of the indication

In relation to our primary outcome, the satisfaction of patients varied depending on the anatomical region of IR. Specifically, patient satisfaction was reported in 47.5% of the patients after IR of the ankle, in 53.6% of the foot, and in 41.2% of the other regions (patella and proximal tibia). We found that patient satisfaction following IR depended on the indication for the procedure. Patient satisfaction increased when there was an objective restriction such as pain (68%) or a combination with limited range of motion (87.5%). Conversely, patients with unspecific symptoms of discomfort had a lower rate of patient satisfaction after IR (27.5%) as demonstrated in Table 2 and Fig. 3.

**Table 2:** Summary of statistical analyses. Odds ratios represent the likelihood of patient satisfaction based on indication and complications based on risk and procedural factors. *CI*, confidence interval

	Odds ratio	Lower 95% CI	Higher 95% CI	*p*-value
Outcome: Improved patient satisfaction				
Pain	6.049	2.979	12.675	0
ROM	1.706	0.513	6.128	0.419
Subjective	0.192	0.092	0.386	< 0.001
Weather sensitivity	3.635	1.041	16.167	0.033
Outcome: Complications				
Smoking	5.178	1.507	20.492	0.006
Comorbidities	0.766	0.170	2.753	0.777
Overdrilling	1.605	0.347	5.947	0.491
Remaining Implants	0	0	16.119	1

**Fig. 4:**
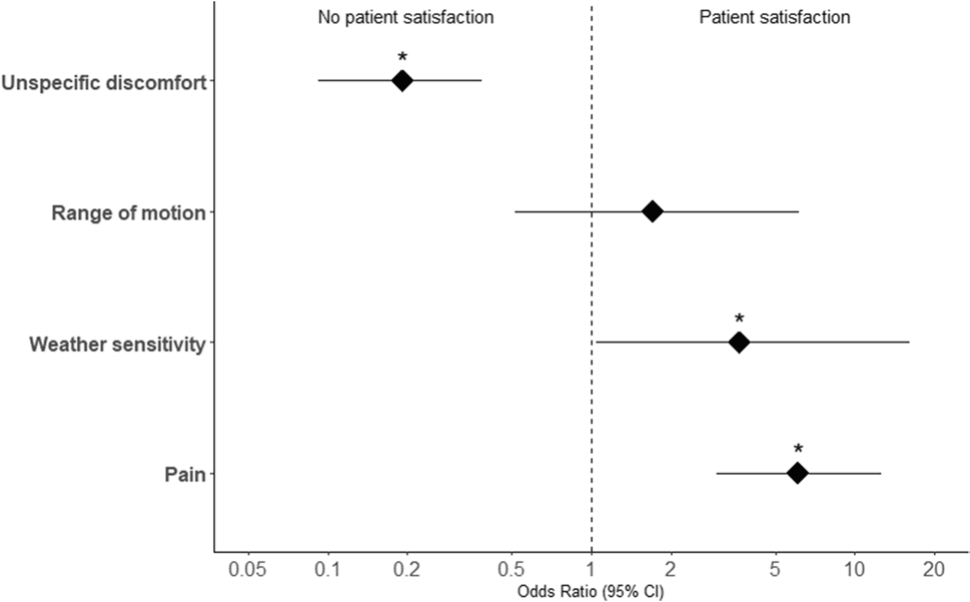
Odds ratios on subjective improvement after implant removal based on individual factors with 95% confidence intervals. *Significant

**Fig. 5:**
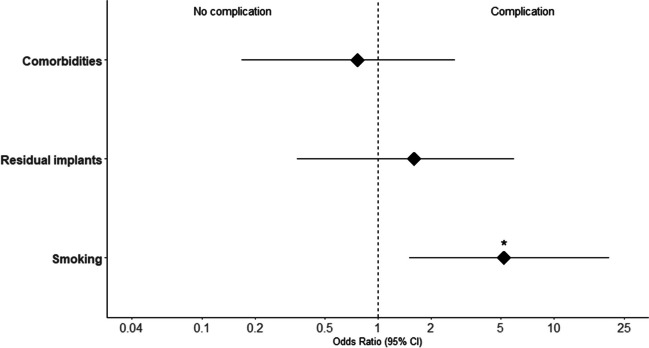
Odds ratios on complications after and during implant removal with 95% confidence intervals. *Significant

Odds ratios were calculated to evaluate the likelihood of patient satisfaction regarding the indication. Patients who preoperatively reported a limited range of motion (OR 1.7, *p* = 0.42) or pain (OR 6.0, *p* = 0) were significantly more likely to experience meaningful relieve of complaints after IR. Patients who only reported unspecific symptoms of discomfort tended to not show a decrease of complaints postoperatively in comparison to all patients (OR 0.19, *p* < 0.001). Patients with a sensitivity to cold weather were also at good odds for relieve of complaints after IR in comparison to patients without (OR 3.6, *p* = 0.03). This is visualized in Table 2, Figs. 4, and  5.

### Secondary outcomes

Minor complications such as wound healing disorders or small bleeding were seen in 8.4%. Major complications occurred in 1.6% of the cases. Overdrilling of damaged implants had to be performed only in 2.8% of the procedures. Refractures after IR were not detected. Persistent pain after IR was detected in 14.7% of the cases (Table 3).

**Table 3:** Intraoperative management and postoperative outcome stratified by body region

	Ankle	Foot	Other
Intraoperative management, *n* (%)			
Overdrilling	3 (2.5)	0 (0.0)	1 (5.9)
Postoperative outcome, *n* (%)			
Patient satisfaction	58 (47.5)	15 (53.6)	7 (41.2)
Improvement ROM	14 (11.5)	6 (21.4)	2 (11.8)
Major complications	2 (1.6)	0 (0)	0 (0)
Deep infections	0 (0)	0 (0)	0 (0)
Refracture	0 (0)	0 (0)	0 (0)
Iatrogenic injuries	2 (1.6)	0 (0)	0 (0)
Minor complications	11 (9.0)	2 (7.1)	1 (5.9)
Superficial infections/wound healing disorders	7 (5.7)	2 (7.1)	1 (5.9)
Persistent pain	15 (12.3)	4 (14.3)	3 (17.6)

Smoking patients showed a significantly higher risk of complications (OR 5.2,* p* = 0.006) (Fig. 5).

## Discussion

Although elective IR in consolidated fractures is commonly performed in orthopedic and trauma surgery, there is ongoing debate regarding its risks and benefits. In addition, there is a lack of guidelines or evidence-based recommendations. Unspecific symptoms of discomfort are a topic of controversy and are frequently cited as a reason for IR. In our study, we examined the outcomes of IR based on different indications, and our main findings were as follows:

- Unspecific symptoms of discomfort are the primary reason for elective IR in the lower extremity.
- Purely subjective complaints are significantly less likely to be relieved after IR.
- While major complications are very rare, we noted a considerable number of minor complications such as wound healing disorders, which were significantly more pronounced in smokers.

Reasons for IR vary and are influenced by the specific anatomical region. Our findings indicated that unspecific symptoms of discomfort were the main drivers for IR. We also identified pain and limited range of motion as significant indications, which sometimes occurred together. Our results align with recent publications. Wurm et al. described limited range of motion, pain, and irritation as major reasons for IR in the lower extremity [7]. Since irritation and symptoms of discomfort are challenging to quantify, standardized assessment of outcomes becomes difficult, potentially leading to patient dissatisfaction. Cold weather pain, prominent implants, and general discomfort are considered additional indications for IR in the literature [4, 8]. However, many studies focused on different anatomical regions, resulting in relatively small sample sizes.

Our second major finding indicated that patients’ satisfaction varied depending on the indication for IR. In our study, only 48.7% of the patients reported subjective benefits after IR when combining all investigated anatomical regions of the lower extremity. Patient satisfaction was lowest when the indication was based on unspecific symptoms of discomfort. We also observed that patient satisfaction increased when the indication was clinically measurable. Therefore, IR based on patient request should be carefully considered. In view of these results, we must reject our initial hypothesis that indications for IR based on unspecific symptoms of discomfort may result in similar patient satisfaction, compared to clinically objective indications, such as pain or limited range of motion.

Several previous studies have discussed the outcomes of elective IRs of the lower extremity. Kempton et al. reported the development of pain in 37.5% of patients after removal of asymptomatic hardware [8]. In our series, we did not observe an increase in complaints after removal of asymptomatic hardware. Wang et al. recommended performing elective IR of healed fractures only upon a patient’s explicit request [9]. A decrease of pain is only described in 50% of symptomatic patients following ankle IR [8]. Considering this, indications for IR in the lower extremity are ambiguous as the goal should always be to provide a benefit for the patient. In addition, there may be a psychological aspect involved in the subjective desire for IR without objective complaints, which should be considered. Furthermore, especially in ankle fractures, overlooked associated pathologies such as syndesmotic instabilities or osteochondral lesions are discussed controversially [10]. Nevertheless, in our study, we observed an increase in patient satisfaction when the indication was clinically measurable, such as pain or a combination of pain and a limited range of motion. Williams et al. described functional improvement and increased range of motion in 73% of lower extremities after IR in a cohort of 85 patients [11, 12]. In our cohort, the most beneficial outcomes were observed after IR on the foot (53.6%). An improvement in range of motion was only detected in 15% of the patients all anatomical regions combined. This gives rise to the question whether these patients would have profited from additional treatments, e.g., an additional arthroscopic arthrolysis [13]. Nevertheless, only 5.8% of the investigated patients reported a limitation of range of motion as leading reason for IR.

Finally, with regard to our secondary outcomes, we demonstrated a low occurrence of postoperative or intraoperative complications in our cohort. The literature describes complication rates ranging from 12 to 41% in elective IR in general and from 5 to 25% in foot and ankle surgery [4, 14]. Furthermore, the procedures are associated with prolonged operative duration and a higher volume of blood loss comparing to the initial procedure [15]. Complications including superficial wound infections, iatrogenic injuries, or refractures were infrequent. Therefore, we consider elective IR to be a relatively safe procedure. Previous studies also described elective IR as a safe procedure with minimal risks [16, 17]. Williams et al. reported a complication rate of 10%, primarily consisting of superficial infections [11]. Suda et al. reported the highest rate of postoperative complications in IR of the calcaneus [18]. Yuan et al. reported no major complications in their study, with only two out of 38 patients complaining about retained material [19]. In our study, we detected impaired wound healing in 7.1% without the occurrence of major complications after IR on the foot. Complications were significantly higher in patients who are smokers. Brown et al. reported on persistent pain after removal of symptomatic implant on the ankle in 8.7% [2]. We were able to show similar results in our study (12.3%). Interestingly, the occurrence of minor complications did not have an influence on the satisfaction rate; however, this might likely be due to the relatively small number.

Finally, there is a financial aspect that hospitals and healthcare providers should consider [15]. While there is no literature directly comparing the costs of elective IR with other orthopedic procedures, one study from the USA highlights that on average elective IR costs approximately $5707, including the surgical procedure and anesthesia [Böstman]. The costs for IR are presumably lower in European countries. Costs of operating rooms without surgeons and anesthesia range from $22 to $133 per minute [20]. Ultimately, there is still the risk that operative procedures based on soft criteria and not based on scientific or evidenced-based indications may not be reimbursed by healthcare providers.

### Strengths

To the best of our knowledge, this study represents the largest cohort of elective IRs in the lower extremity reported in the literature. Additionally, our study focused on the three most operated regions of the lower extremity, resulting in a highly homogeneous patient group. All patients underwent regular preoperative evaluation, as well as follow-up visits on the first day postoperatively and after 6 weeks. This ensured detailed documentation and patient information, enabling a comparable assessment of the investigated parameters. Moreover, we placed particular emphasis on discussing potential subjective complaints with all patients, an aspect that is often overlooked in recent literature that predominantly focuses on objective parameters.

### Limitations

This study is a retrospective cohort study. Only patients who completed a follow-up of at least 6 weeks after surgery were included, while those without completed follow-ups were excluded. As a result, patients who experienced positive outcomes after IR may not have attended the scheduled appointments, leading to an underrepresentation of patients benefiting from the procedure and introducing a selection bias. Furthermore, we did not compare the long-term outcome of patients who underwent elective IR with a control group without IR. Complaints that arose more than 6 weeks after IR surgery were also not captured. Another limitation is the subjective nature of patient satisfaction. We solely focused on patient satisfaction or dissatisfaction and clinical examinations in the postoperative course, without using specific scoring systems for measurement.

## Conclusion

The results of our study underline the significance of patients’ education when the indication for hardware removal is based on unspecific symptoms of discomfort. In these patients, dissatisfaction with the outcome of IR was the highest and frustrating persistence of preoperative symptoms was often reported. In addition, we were able to detect a risk for minor complications after IR of the lower extremity. We believe that routine IR in asymptomatic patients with lower extremity fractures should not generally be recommended and should only be considered after providing detailed education to patients about potential complication outcome. Our study confirms a low occurrence of intra- and postoperative risks. The psychological aspect of strong subjective request for elective IR should be further investigated.

In cases where IR is based on unspecific symptoms of discomfort or purely cosmetic reasons, it is crucial to educate the patient about potential risks. These factors may modify patients’ expectations and should be considered before recommending or performing the procedure.

## Abbreviations

CI: Confidence interval
COM: Complications
IR: Implant removal
OR: Odds ratio
SD: Standard deviation
SI: Subjective improvement
ROH: Removal of hardware
ROM: Range of motion
